# Supplementary figures and images for: Peach-Morchella intercropping mode affects soil properties and fungal composition
Source: PeerJ. 2021 Jul 12;9:e11705. doi: 10.7717/peerj.11705 (PMC8280869; doi:10.7717/peerj.11705)

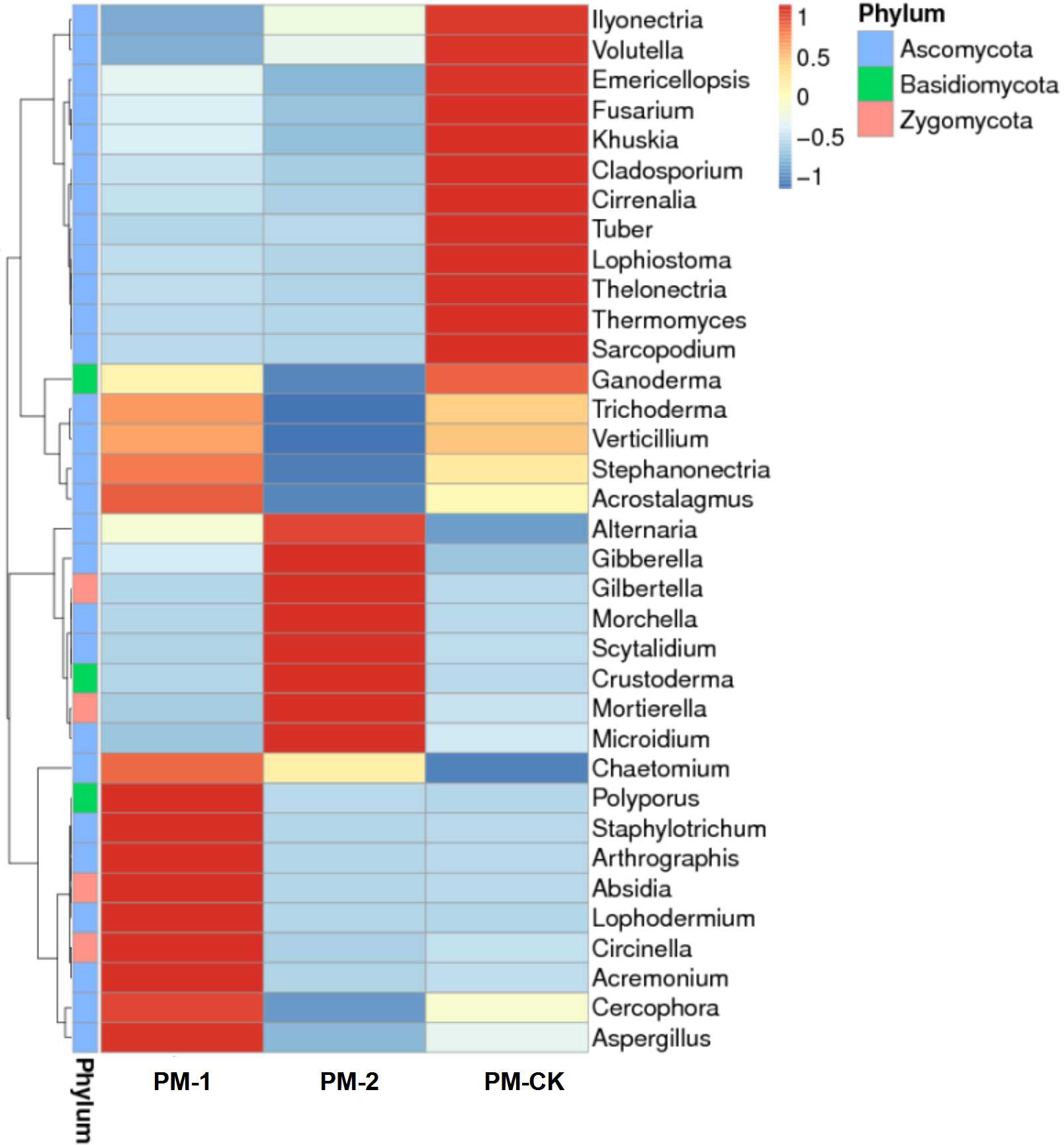

Supplement: Supplemental Information 3 [file peerj-09-11705-s003.jpg]

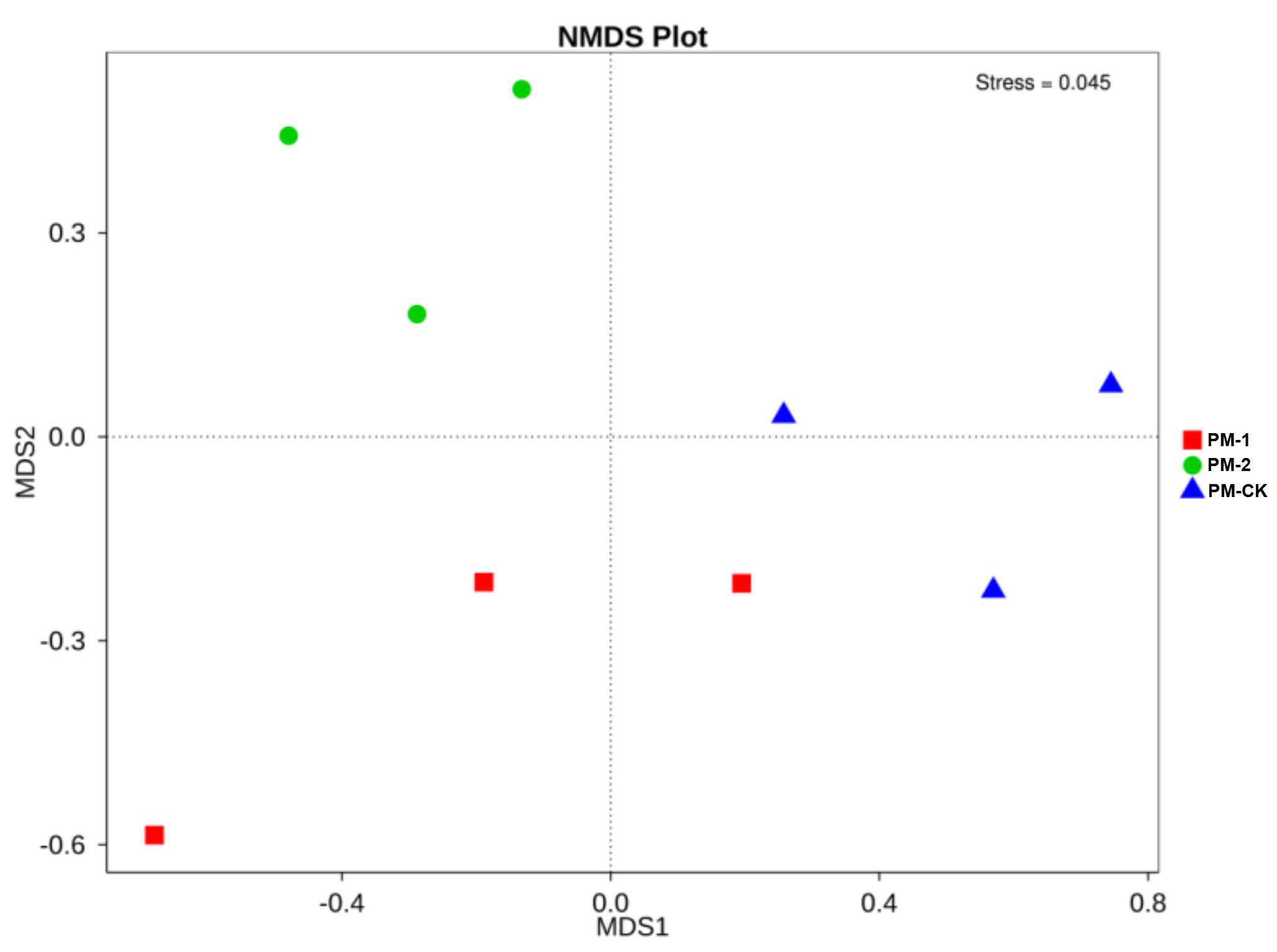

Supplement: Supplemental Information 4 — Each point in the diagram represents a fungal community sample. The closer distance between two points in the ordination space indicates the lower dissimilarity between the microbial community structure of these two samples. [file peerj-09-11705-s004.jpg]

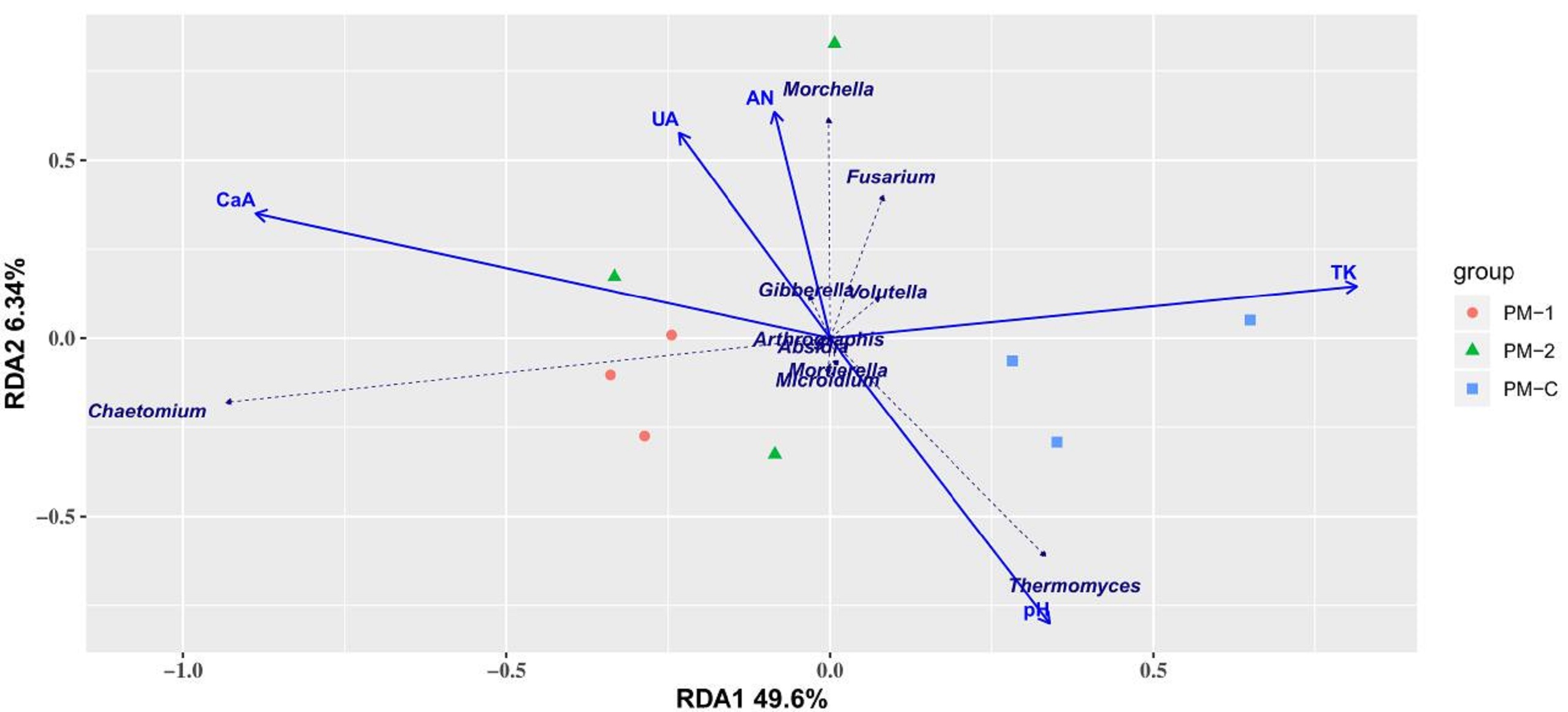

Supplement: Supplemental Information 5 — CaA, catalase activity; UA, urease activity; AN, available nitrogen; TK, total potassium; pH, pH value. [file peerj-09-11705-s005.jpg]
